# Inhibition of Pyramidal Neurons in the Basal Amygdala Promotes Fear Learning

**DOI:** 10.1523/ENEURO.0272-18.2018

**Published:** 2018-10-31

**Authors:** Megan Tipps, Ezequiel Marron Fernandez de Velasco, Allee Schaeffer, Kevin Wickman

**Affiliations:** Department of Pharmacology, University of Minnesota, Minneapolis, Minnesota 55455

**Keywords:** basal amygdala, DREADD, fear conditioning, inhibitory signaling, learning and memory

## Abstract

The basolateral amygdala complex, which contains the lateral (LA) and basal (BA) subnuclei, is a critical substrate of associative learning related to reward and aversive stimuli. Auditory fear conditioning studies in rodents have shown that the excitation of LA pyramidal neurons, driven by the inhibition of local GABAergic interneurons, is critical to fear memory formation. Studies examining the role of the BA in auditory fear conditioning, however, have yielded divergent outcomes. Here, we used a neuron-specific chemogenetic approach to manipulate the excitability of mouse BA neurons during auditory fear conditioning. We found that chemogenetic inhibition of BA GABA neurons, but not BA pyramidal neurons, impaired fear learning. Further, either chemogenetic stimulation of BA GABA neurons or chemogenetic inhibition of BA pyramidal neurons was sufficient to generate the formation of an association between a behavior and a neutral auditory cue. This chemogenetic memory required presentation of a discrete cue, and was not attributable to an effect of BA pyramidal neuron inhibition on general freezing behavior, locomotor activity, or anxiety. Collectively, these data suggest that BA GABA neuron activation and the subsequent inhibition of BA pyramidal neurons play important role in fear learning. Moreover, the roles of inhibitory signaling differ between the LA and BA, with excitation of pyramidal neurons promoting memory formation in the former, and inhibition of pyramidal neurons playing this role in the latter.

## Significance Statement

The basolateral amygdala complex, which consists of lateral (LA) and basal (BA) subnuclei, is a critical substrate of associative learning. Although inhibition of GABAergic interneurons and subsequent disinhibition of pyramidal neurons in the LA is critical to fear learning, the contribution of the BA is less clear. Here, we used a chemogenetic approach to manipulate pyramidal and GABA neuron excitability in the BA during fear conditioning. We found that BA GABA neuron activity is necessary for fear learning, and that BA GABA neuron stimulation or BA pyramidal neuron inhibition can induce an association between a behavior and an auditory cue. These findings expand our understanding of the fear learning circuitry and highlight a novel role for inhibitory signaling.

## Introduction

Abnormal associative learning is a hallmark of many mental health disorders, including obsessive compulsive disorder, post-traumatic stress disorder, and drug addiction (Nielen et al., 2009; Maren et al., 2013; Bowers and Ressler, 2015; Taylor and Torregrossa, 2015). These disorders are characterized by the aberrant establishment of associations between two stimuli or a stimulus and behavior, or the failure to extinguish associations that are no longer relevant. This can result in exaggerated or inappropriate emotional responses, as well as prolonged maladaptive behaviors that persist despite negative consequences. Understanding the neuronal mechanisms that support the formation and durability of associative memories is key to developing effective therapies for diseases in which associative learning is disrupted.

The amygdala has been studied extensively for its role in the formation and extinction of emotional memories and motivated behaviors (LeDoux, 2000; Janak and Tye, 2015). Much of what is known regarding the mechanisms within the amygdala that underlie associative learning derives from Pavlovian fear conditioning studies (Maren, 2001; Fanselow and Poulos, 2005; Pape and Pare, 2010; Johansen et al., 2011; Maren et al., 2013). Fear conditioning is a task in which an aversive unconditioned stimulus [(US) such as a footshock], is paired with a neutral conditioned stimulus [(CS) often an auditory cue], leading to a learned association between the US and CS (Izquierdo et al., 2016). Following fear conditioning, presentation of the originally neutral CS evokes a fearful response. Fear conditioning studies in both animal models and humans have consistently highlighted the importance of the amygdala to the formation and extinction of fear memories (LeDoux, 2000; Pape and Pare, 2010; Johansen et al., 2011; Janak and Tye, 2015), including identifying specific roles for the various subnuclei of the amygdala in these processes (Duvarci and Pare, 2014; Herry and Johansen, 2014).

The basolateral amygdala (BLA) complex is a key input region of the amygdala and a critical substrate of fear memory formation (Duvarci and Pare, 2014; Janak and Tye, 2015). The BLA complex contains lateral (LA) and basal (BA) subdomains (Sah et al., 2003), and data suggest that each domain makes distinct contributions to fear learning. The LA is a node of convergence for thalamic and sensory input related to conditioned and unconditioned stimuli (LeDoux et al., 1990; Romanski et al., 1993; McDonald, 1998). Lesions of the LA disrupt fear learning (LeDoux et al., 1990; Amorapanth et al., 2000; Goosens and Maren, 2001; Nader et al., 2001; Calandreau et al., 2005), and targeted optogenetic studies have shown that LA pyramidal neuron excitation is a critical step in the acquisition of fear memories (Johansen et al., 2010; Yiu et al., 2014). Moreover, the level of excitability of LA pyramidal neurons correlates with the likelihood that they are included in the fear memory trace (Zhou et al., 2009; Kim et al., 2014). Interestingly, the strength of local inhibitory input to LA pyramidal neurons bi-directionally regulates the strength of fear memories. Indeed, the disinhibition of LA pyramidal neurons, driven by the US-mediated inhibition of parvalbumin interneurons, has been implicated in the gating of fear memory formation (Wolff et al., 2014; Letzkus et al., 2015).

The BA receives glutamatergic input from the LA (Krettek and Price, 1978; Smith and Paré, 1994), as well as from the ventral hippocampus (vHPC) and medial prefrontal cortex (mPFC; Maren et al., 2013; Zelikowsky et al., 2014; Rozeske et al., 2015; McDonald and Mott, 2017). Although connections with the vHPC and mPFC are consistent with its role in contextual fear learning and in the expression of fear memories (Goosens and Maren, 2001; Calandreau et al., 2005; Amano et al., 2011; Akagi Jordão et al., 2015), the relevance of the BA to the acquisition of fear memories is less clear (Goosens and Maren, 2001; Nader et al., 2001; Anglada-Figueroa and Quirk, 2005). To address this issue, we used a neuron-specific chemogenetic approach to probe the relevance of BA pyramidal and GABA neurons to auditory fear conditioning. Our data show that BA GABA neuron activity is necessary for auditory fear conditioning, and that either the stimulation of BA GABA neurons or inhibition of BA pyramidal neurons is sufficient to induce an association between a behavior and a neutral auditory cue.

## Materials and Methods

### Animals

Animal experiments were approved by the University of Minnesota Institutional Animal Care and Use Committee. CaMKIICre [B6.Cg-Tg(Camk2a-cre)T29-1Stl/J] and GADCre (B6N.Cg-*Gad2^tm2(cre)Zjh^*/J) lines were purchased from The Jackson Laboratory, and have been maintained by backcrossing against the C57BL/6J strain. Offspring from these crosses were used to generate the Cre(+) and Cre(−) mice used in this study. The generation of conditional CaMKIICre(+):*Girk1^fl/fl^* mice was described previously (Marron Fernandez de Velasco et al., 2017). Unless specifically noted, males and females were used in all experiments, and groups were balanced by sex. All mice were maintained on a 12 h light/dark cycle, and were provided ad libitum access to food and water.

### Reagents

Picrotoxin (PTX), kynurenic acid, and barium chloride were purchased from Sigma-Aldrich. Clozapine-n-oxide (CNO) and tetrodotoxin (TTX) were purchased from Tocris Bioscience.

### Intracranial viral manipulations

AAV8-hSyn-DIO-hM4Di-mCherry and AAV8-hSyn-DIO-hM3Dq-mCherry were purchased from the UNC Vector Core. Mice (7-8 weeks old) were placed in a stereotaxic device (David Kopf Instruments) under isoflurane anesthesia. Microinjectors were made by affixing a 33-gauge stainless steel hypodermic tube within a shorter 26-gauge stainless steel hypodermic tube. The microinjectors were attached to polyethylene-20 tubing affixed to 10 μl Hamilton syringes, and were lowered through burr holes in the skull to the BA (from bregma: −1.65 mm A/P, ±3.25 mm M/L, −4.7 mm D/V) or LA (from bregma: −1.6 mm A/P, ±3.3 mm M/L, −4.2 mm D/V); 500 nl (4–7 × 10^12^ viral particles/ml) of viral solution per side was injected over 5 min. The syringe was left in place for 10 min following infusion to reduce solution backflow along the infusion track. Subsequent electrophysiological and behavioral experiments were performed 4 weeks after surgery to allow for full recovery and viral expression. The scope and accuracy of viral targeting was assessed by tracking viral-mediated mCherry fluorescence in serial coronal sections Cre(+) mice. Fluorescence was observed along the full rostrocaudal axis of the BLA complex. Only data from mice in which the majority (>80%) of expression was confined to the targeted subregion (LA or BA), with limited or no diffusion to adjacent structures (i.e., central amygdala or cortex), were analyzed.

### Slice electrophysiology

Coronal slices (270–280 μm) containing the BLA complex were prepared from mice (5–12 weeks), as described previously (Arora et al., 2010; Hearing et al., 2013; Marron Fernandez de Velasco et al., 2017), and were incubated at 32°C in ACSF for >30 min before recording. All measured and command potentials factored in a junction potential (−15 mV) predicted using JPCalc software (Molecular Devices). Agonist-induced somatodendritic currents were measured in an ACSF bath using a K-gluconate pipette solution, at a holding potential (*V*_hold_) of −60 mV. Holding current, input resistance, and series resistance values were monitored during each experiment by tracking responses to periodic (0.2 Hz) voltage steps (−5 mV, 800 ms). Only experiments with stable (<20% variation) and low series resistances (<30 MΩ) were analyzed. For rheobase assessments, cells were held in current-clamp mode and given 1 s current pulses, beginning at −60 pA and progressing in 20 pA increments until spiking was elicited. Miniature IPSCs (mIPSCs) were recorded (V_hold_= −70 mV) for 1.5 min using a 140 mm CsCl-based pipette solution, with 2 mm kynurenic acid and 0.5 μm TTX present in the bath to block ionotropic glutamatergic activity and action potentials, respectively. mIPSCs were analyzed with Minianalysis software (Synaptosoft), using a 10 pA detection threshold. All electrophysiological datasets include data from at least two offspring from different breeder pairs.

### Behavioral testing

Adult mice (8–12 weeks) were evaluated using established delay fear conditioning (Tipps et al., 2014) and elevated plus maze (EPM) tasks (Victoria et al., 2016). For fear conditioning experiments, the CS was a 65 dB white noise (30 s) and the US was a 0.5 mA footshock (2 s), administered during the last 2 s of the CS presentation. Before training, mice were exposed to the fear conditioning room and pre-handled for 2 d to acclimate animals to the behavioral room and investigator. Four conditioning protocols were used in this study: (1) 3 CS/3 US: mice were exposed to three CS–US pairings, with CS presentations separated by 90 s (7.5 min total); (2) 3 CS/0 US: mice were exposed to the CS three times using the same timing sequence as with the 3 CS/3 US protocol, but no US was delivered (7.5 min total); (3) 0 CS/0 US: mice were placed in the chambers but were not exposed to either CS or US (7.5 min total); and (4) 1 CS/1 US: mice were exposed to a single CS–US pairing, preceded and followed by 90 s intervals (3.5 min total). To assess context learning (24 h after training), mice were returned to the conditioning chambers and freezing was evaluated for 5 min. To assess cue learning (48 h after training), chambers were reconfigured using a white plastic insert to cover the bar floor and a black tent insert to alter the size, shape, and color of the chambers. Inserts were cleaned with 0.1% acetic acid to provide a distinct olfactory cue. Freezing was monitored throughout the 15 min cue recall test period, divided into 5 × 3 min bins that included 2 × 3 min CS presentations (Tipps et al., 2014). Freezing behavior, along with measures of average and maximum motion, was assessed automatically using Video Freeze v2.6.1.72 software (Med Associates). For EPM studies, time spent in open and closed arms, and number of arm entries, were recorded for 5 min, beginning when the mouse made its first entry into any arm. EPM performance was scored manually, by an experienced investigator.

### Data analysis

Data are presented throughout as the mean ± SEM. Statistical analyses were performed using Prism 6 (GraphPad Software) and SigmaPlot 11.0 (Systat Software). Sex was included as a variable in initial analyses. Because no impact of sex was observed in any study, all data from male and female subjects were pooled. Pooled data were analyzed by Student’s *t* test or repeated-measures ANOVA, as appropriate. Pairwise comparisons were performed using Bonferroni or Holms–Sidak (H–S) tests, when appropriate. For all statistical comparisons, differences were considered significant if *p* < 0.05.

## Results

### Chemogenetic inhibition of BA GABA neurons impairs auditory fear conditioning

**Figure 1.**
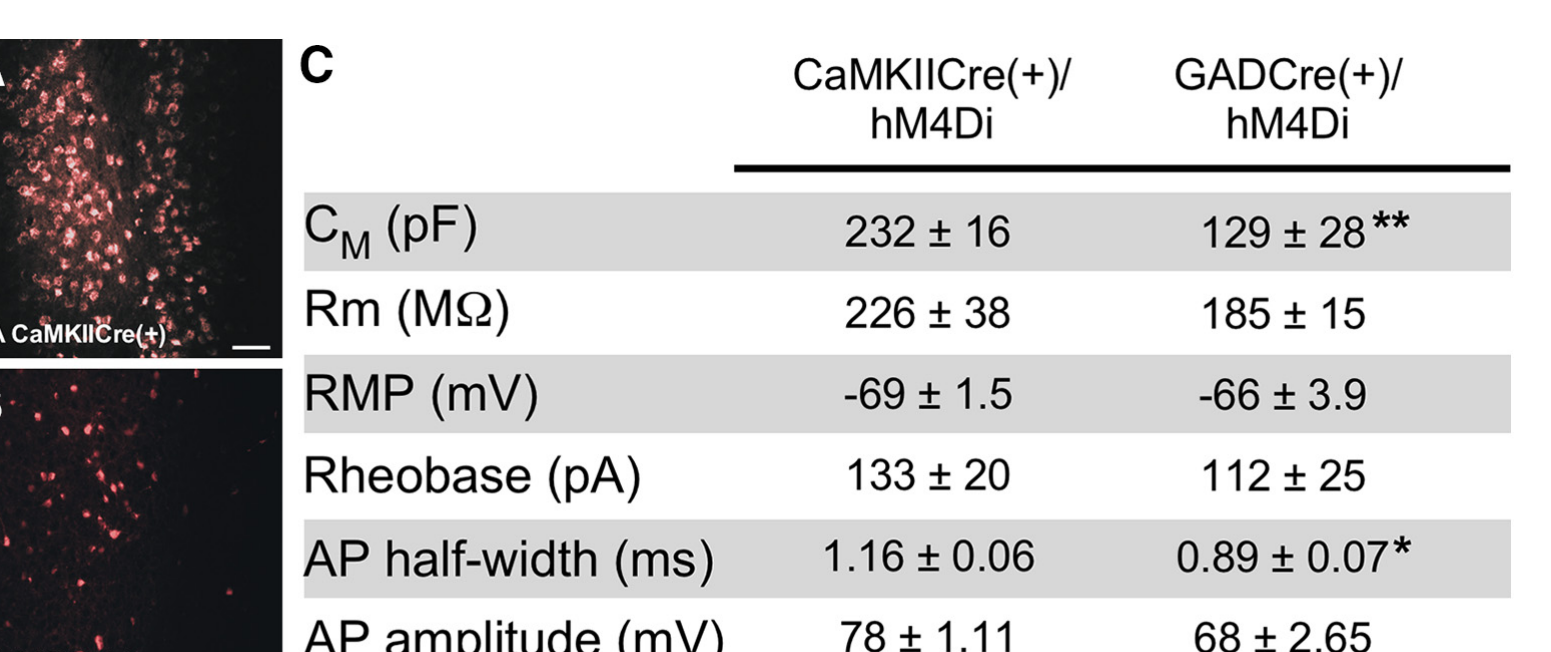
Chemogenetic manipulation of BA pyramidal and GABA neurons. ***A***, ***B***, hM4Di-mCherry fluorescence in the BA of CaMKIICre(+) and GADCre(+) mice, 4 weeks after infusion of AAV8-hSyn-DIO-hM4Di-mCherry. Scale bar, 20 μm. ***C***, Summary of intrinsic and active electrophysiological properties of hM4Di-expressing BA neurons from CaMKIICre(+) and GADCre(+) mice, 4 weeks after intra-BA infusion of AAV8-hSyn-DIO-hM4Di-mCherry. Apparent capacitance (C_M_), input/membrane resistance (Rm), and resting membrane potential (RMP) were extracted on achieving whole-cell access. Action potential (AP) characteristics, including threshold (rheobase), half-width, and amplitude, were extracted from the initial spike evoked at rheobase. hM4Di-expressing neurons from GADCre(+) mice had significantly smaller membrane capacitance (*t*_(15)_=3.3, **p = 0.005) and AP half-widths (*t*_(15)_=2.9, *p = 0.01) compared to hM4Di-expressing neurons from CaMKIICre(+) mice (*n* = 8–15 neurons/group, derived from at least 3 mice/group).

The BLA complex contains two primary neuron types: the more abundant (85%) glutamatergic pyramidal neurons that project to several brain regions (Sah et al., 2003; Pape and Pare, 2010), and the less abundant (15%) GABA interneurons that regulate the excitability of pyramidal neurons (Spampanato et al., 2011). We used a transgenic Cre approach and a Cre-dependent inhibitory chemogenetic viral vector (AAV8-hSyn-DIO-hM4Di-mCherry) to facilitate neuron-specific chemogenetic inhibition of BA pyramidal or GABA neurons. To drive Cre-dependent expression of hM4Di in pyramidal neurons, we used the well characterized CaMKIICre transgenic mouse line, which promotes Cre-dependent recombination in pyramidal neurons in many brain regions (Tsien et al., 1996; Sonner et al., 2005; Madisen et al., 2010; Victoria et al., 2016), including pyramidal neurons in the BLA complex (Wolff et al., 2014; Marron Fernandez de Velasco et al., 2017). To target BA GABA neurons, we used GADCre mice, in which Cre-dependent recombination is evident in all major interneuron subtypes throughout the central nervous system (Taniguchi et al., 2011). Visual comparison of viral-driven fluorescence revealed that AAV8-hSyn-DIO-hM4Di-mCherry treatment highlighted a less abundant neuron population, with smaller somata, in GADCre(+) compared to CaMKIICre(+) mice (Fig. 1*A***,***B*). Consistent with published reports (Rainnie et al., 1993; Sosulina et al., 2006; Bocchio et al., 2015), hM4Di-positive neurons in slices from GADCre(+) mice (putative GABA neurons) had significantly smaller apparent capacitances and shorter action potential half-widths than hM4Di-positive neurons from CaMKIICre(+) mice (putative pyramidal neurons; Fig. 1*C*). Thus, CaMKIICre(+) and GADCre(+) mice permit the targeted manipulation of distinct populations of neurons in the BA, with morphologic and electrophysiological properties consistent with those reported previously for BLA complex pyramidal and GABA neurons, respectively.

**Figure 2.**
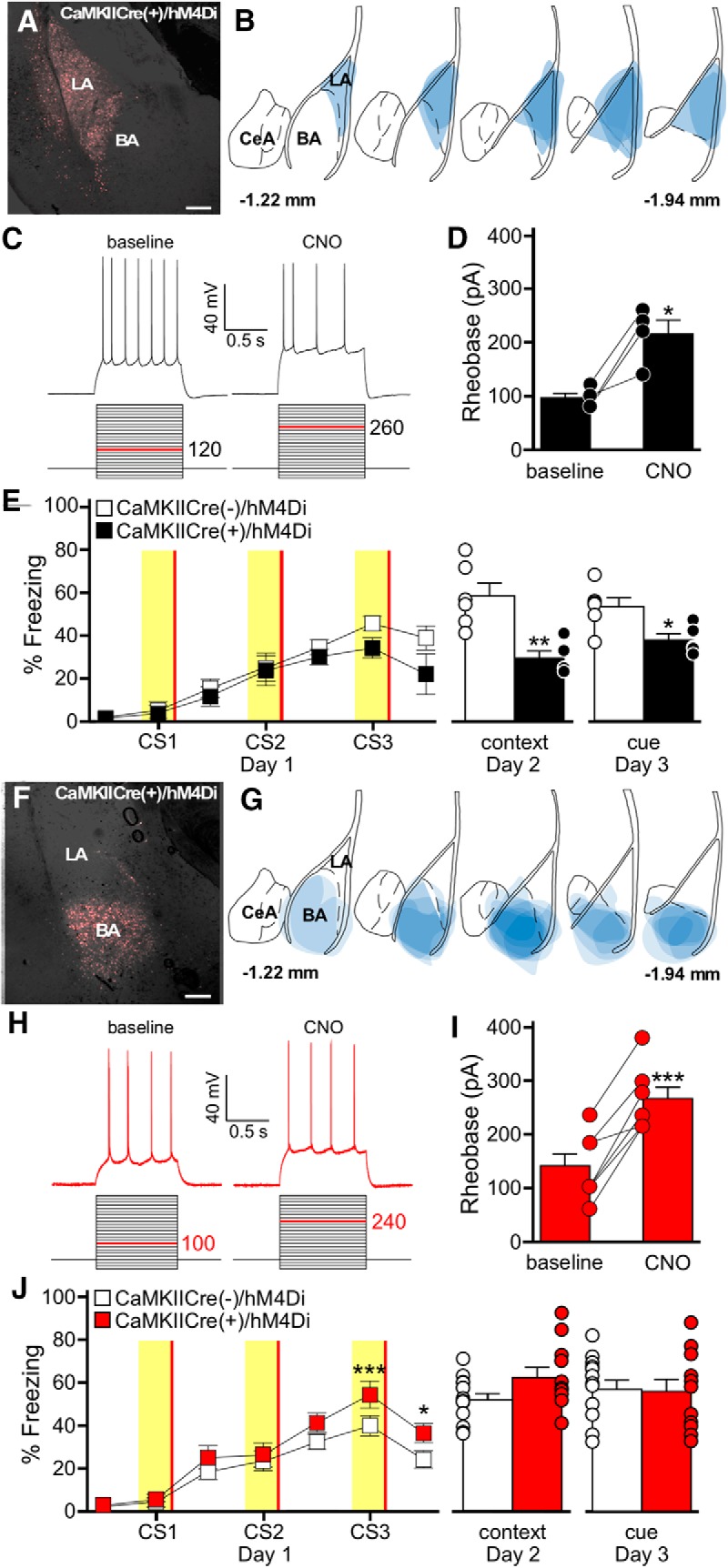
Chemogenetic inhibition of pyramidal neurons in the LA, but not BA impairs auditory fear conditioning. ***A***, hM4Di-mCherry fluorescence in the LA of a CaMKIICre(+) mouse, 4 weeks after infusion of AAV8-hSyn-DIO-hM4Di-mCherry. Scale bar, 50 μm. ***B***, Schematic summarizing the distribution of hM4Di-mCherry fluorescence in the LA (spanning –1.22mm to –1.94mm posterior from bregma) of CaMKIICre(+) mice evaluated in ***E***. ***C***, CNO-induced inhibition of an hM4Di-expressing LA pyramidal neuron. Rheobase was measured before (baseline) and after application of CNO (10 μm). The current-step protocol is depicted below the traces. The first step to elicit spiking is highlighted in red; the trace shown is the response to the denoted current step. ***D***, Rheobase summary for hM4Di-expressing LA pyramidal neurons, before and after CNO application (*t*_(3)_=4.4, *p = 0.021). Each experiment is shown as connected circles (*n* = 4). ***E***, Impact of LA pyramidal neuron inhibition on auditory fear conditioning. CaMKIICre(+)/hM4Di (black) and CaMKIICre(−)/hM4Di (white) mice were trained using a 3 CS/3 US paradigm. CNO (2 mg/kg, i.p.) was administered 30 min before training. Freezing behavior during training is plotted on the left. The yellow bars represent CS presentations, and the red bars represent US presentations. There was no main effect of genotype (*F*_(1,54)_=2.6, *p* = 0.14). The plots on the right show freezing during context (*t*_(9)_=4.0, **p = 0.003) and cue (*t*_(9)_=2.9, *p = 0.017) recall tests. The bars represent the mean ± SEM, with dots next to the bars denoting individual data points (*n* = 5–6/group). Sex differences were not assessed for this experiment. ***F***, hM4Di-mCherry fluorescence in the BA of a CaMKIICre(+) mouse, 4 weeks after infusion of AAV8-hSyn-DIO-hM4Di-mCherry. Scale bar, 50 μm. ***G***, Schematic summarizing the distribution of hM4Di-mCherry fluorescence in the BA (coronal view, spanning –1.22mm to –1.94mm posterior from bregma) of CaMKIICre(+)/hM4Di mice evaluated in ***C***. ***H***, CNO-induced inhibition of an hM4Di-expressing BA pyramidal neuron. Rheobase was measured before (baseline) and after application of CNO (10 μm), as described for ***C***. ***I***, Rheobase summary for hM4Di-expressing BA pyramidal neurons, before and after CNO application (*t*_(6)_=7.6, ***p < 0.001). Each experiment is shown as connected circles (*n* = 7). ***J***, Impact of BA pyramidal neuron inhibition on fear learning. CaMKIICre(+)/hM4Di (red) and CaMKIICre(−)/hM4Di (white) mice were trained using a 3 CS/3 US paradigm. CNO (2 mg/kg, i.p.) was administered 30 min before training. Freezing behavior during training is plotted on the left. The yellow bars represent CS presentations, and the red bars represent US presentations. There was a significant main effect of genotype (*F*_(1,132)_=4.9, *p* = 0.038; CS3: ***p < 0.001, post-CS3: *p = 0.023). The plots on the right show freezing during context (*t*_(22)_=1.9, *p* = 0.074) and cue (*t*_(29)_=0.1, *p* = 0.905) tests. Error bars represent the mean ± SEM, with dots next to the bars denoting individual data points (*n* = 5/6–7/5 males/females per group).

To test the efficacy of the neuron-specific chemogenetic approach, we first examined whether chemogenetic inhibition of LA pyramidal neurons could disrupt auditory fear learning. Previous work has shown that optogenetic inhibition of LA pyramidal neurons disrupted auditory fear conditioning (Johansen et al., 2014), a key line of evidence supporting the contention that LA pyramidal neuron excitation is critical to fear learning. We targeted the LA of CaMKIICre(+) mice with an AAV8-hSyn-DIO-hM4Di-mCherry virus, and observed robust hM4Di expression 4 weeks later (Fig. 2*A*,*B*). Application of the hM4Di agonist CNO (10 μm) to acutely isolated slices from these mice reduced the excitability (i.e., increased the rheobase) of hM4Di-expressing LA pyramidal neurons (Fig. 2*C*,*D*). We next evaluated viral-treated CaMKIICre(+) and CaMKIICre(-) littermates in an amygdala-dependent delay fear conditioning protocol involving three pairings of an auditory cue/CS and footshock/US (3 CS/3 US; Fanselow and LeDoux, 1999; LeDoux, 2000; Nonaka et al., 2014). All subjects received CNO (2 mg/kg, .i.p.) 30 min before training, to promote inhibition of hM4Di-expressing neurons during the early (acquisition and consolidation) stages of fear learning (Roth, 2016). Context and cue recall testing occurred 24 and 48 h after training, respectively, in the absence of CNO. All subjects received the same viral and CNO treatments, and because CNO was only administered before training, any phenotypes observed during recall tests were interpreted as reflecting an impact of the neuron-specific manipulation on long-term fear memory formation. As predicted based on previous optogenetic inhibition experiments (Johansen et al., 2014), chemogenetic inhibition of LA pyramidal neurons during training impaired fear memory formation, as evidenced by decreased freezing during both the context and cue recall tests (Fig. 2*E*). Using the same approach to target BA pyramidal neurons in CaMKIICre(+) mice (Fig. 2*F*,*G*), we observed that CNO also reduced the excitability (increased the rheobase) of hM4Di-expressing BA pyramidal neurons (Fig. 2*H*,*I*). Chemogenetic inhibition of BA pyramidal neurons. Although the CaMKIICre(+) mice showed increased freezing to the third CS presentation during training, there was no difference in recall for either the auditory cue or associated context in the 3 CS/3 US paradigm (Fig. 2*J*). Thus, inhibition of BA pyramidal neurons during training did not impair long-term memory formation.

**Figure 3.**
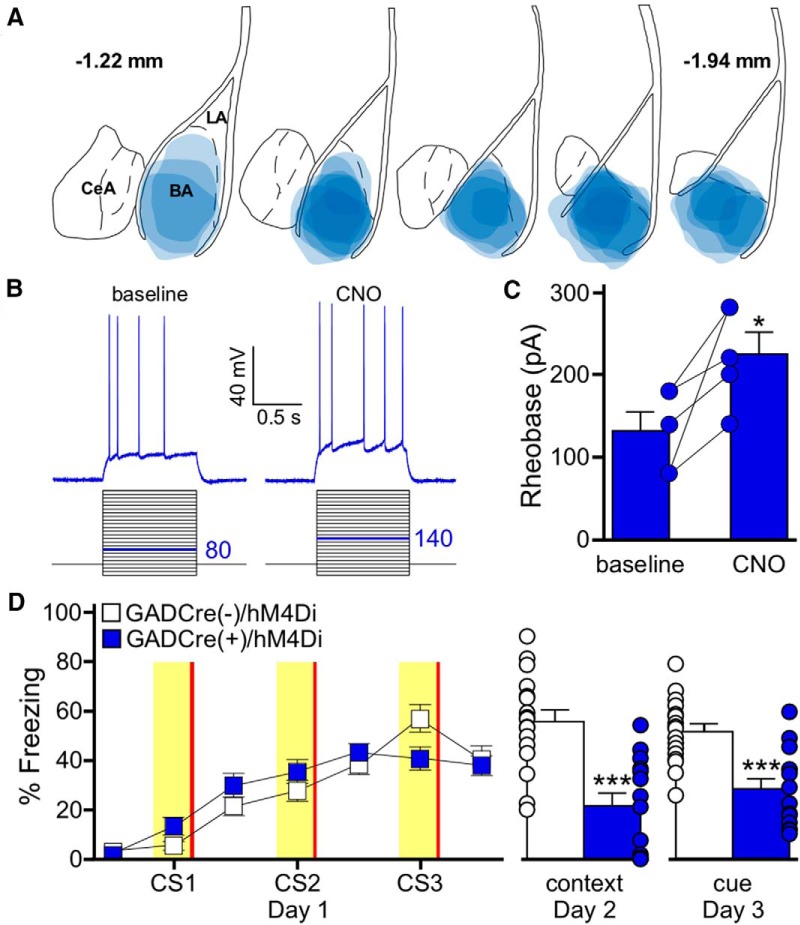
Chemogenetic inhibition of BA GABA neurons impairs auditory fear conditioning. ***A***, Schematic summarizing the distribution of hM4Di-mCherry fluorescence in the BA (coronal view, spanning –1.22mm to –1.94mm posterior from bregma) of GADCre(+)/hM4Di mice evaluated in ***D***. ***B***, CNO-induced inhibition of an hM4Di-expressing BA GABA neuron. Rheobase was measured before (baseline) and after application of CNO (10 μm). The current-step protocol is depicted below the traces. The first step to elicit spiking is highlighted in blue; the trace shown is the response to the denoted current step. ***C***, Rheobase summary for hM4Di-expressing BA GABA neurons, before and after CNO application (*t*_(4)_=3.2, *p = 0.033). Each experiment is shown as connected circles (*n* = 5). ***D***, Impact of BA GABA neuron inhibition on fear learning. GADCre(+)/hM4Di (blue) and GADCre(−)/hM4Di (white) mice were trained with a 3 CS/3 US paradigm. CNO (2 mg/kg, i.p.) was given 30 min before training. Freezing during training is shown on the left. The yellow bars denote CS presentations and the red bars indicate US presentations. There was no main effect of genotype (*F*_(1,174)_=0.1, *p* = 0.71). The plots on the right show freezing during context (*t*_(29)_=4.9, ***p < 0.001) and cue (*t*_(29)_=4.5,***p < 0.001) tests. Error bars represent the mean ± SEM, with dots next to the bars denoting individual data points (*n* = 6/8–9/8 males/females group).

The ability of LA pyramidal neuron inhibition to impair memory formation is consistent with previous reports; however, the lack of effect of BA pyramidal neuron inhibition on fear learning suggests that inhibitory signaling may play a different role in this region. To probe the impact of local GABA-mediated inhibitory signaling within the BA on fear learning, we expressed hM4Di in the BA of GADCre(+) mice (Fig. 3*A*). As expected, CNO reduced the excitability (increased the rheobase) of hM4Di-expressing BA GABA neurons in slices from GADCre(+) subjects (Fig. 3*B*,*C*). In contrast to our results in BA pyramidal neurons, chemogenetic inhibition of BA GABA neurons during training significantly impaired fear memory formation, as illustrated by reduced freezing during the context and cue recall tests (Fig. 3*D*). Thus, GABA neuron activity in the BA is required for the acquisition of fear memory.

### Chemogenetic stimulation of BA GABA neurons promotes fear learning

**Figure 4.**
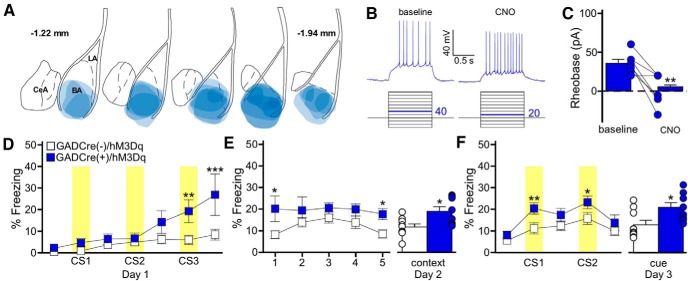
BA GABA neuron stimulation generates an association between a behavior and an auditory cue. ***A***, Schematic summarizing the distribution of hM3Dq-mCherry fluorescence in the BA (coronal view, spanning –1.22mm to –1.94mm posterior from bregma) of the GADCre(+)/hM3Dq mice evaluated in ***D***–***F***. ***B***, CNO-induced stimulation of an hM3Dq-expressing BA GABA neuron. Rheobase was measured before (baseline) and after application of CNO (10 μm). The current-step protocol is depicted below the traces. The first step to elicit spiking is highlighted in blue; the trace shown is the response to the denoted current step. ***C***, Rheobase summary for hM3Dq-expressing BA GABA neurons, before and after CNO application (*t*_(6)_=4.4, **p < 0.001). Each experiment is represented as connected circles (*n* = 7). ***D***, Impact of BA GABA neuron stimulation during training. Freezing behavior during training (3 CS/0 US) for GADCre(+)/hM3Dq (blue) and GADCre(−)/hM3Dq (white) mice. CNO (2 mg/kg, i.p.) was given 30 min before training. Yellow bars represent CS presentations. There was a significant main effect of genotype (*F*_(1,90)_=6.5, *p* = 0.020; CS3: **p = 0.005, post-CS3: ***p < 0.001). ***E***, Freezing during the context recall test. The plot on the left shows freezing for each 1 min bin of the 5 min context test. There was a significant main effect of genotype (*F*_(1,60)_=7.9, *p* = 0.013; bin 1: *p = 0.01, bin 5: *p = 0.04). The plot on the right shows the mean percentage freezing for the entire context test (*t*_(15)_= −2.8, *p = 0.013). ***F***, Freezing during the cue recall test. The plot on the right shows freezing during each 3 min bin of the cue test, with CS presentations highlighted in yellow. There was a significant main effect of genotype (*F*_(1,75)_=13.6, *p* < 0.001; CS1: **p = 0.008, CS2: *p = 0.03). GADCre(+)/hM3Dq (*t*_(14)_= −2.6, p = 0.021), but not GADCre(−)/hM3Dq mice (*t*_(16)_=−1.5, *p* = 0.152) froze significantly more during the CS periods than the non-CS periods. The bars on the right represent mean percentage freezing during the combined CS presentations (*t*_(15)_=−2.5, *p = 0.023). Error bars in ***C***, ***E***, and ***F*** represent the mean ± SEM, with dots next to the bars denoting individual data points (*n* = 4/4–5/4 males/females per group).

Previous work has shown that direct optogenetic stimulation of LA pyramidal neurons during presentation of a neutral auditory cue, in the absence of a footshock, is sufficient to generate a fear-like response to subsequent presentation of the cue (Johansen et al., 2010). Given the similar disruptive impact of chemogenetic inhibition of LA pyramidal (Fig. 2*E*) and BA GABA neurons (Fig. 3*D*) on fear learning, we next asked whether BA GABA neuron stimulation could also generate a learned response to an auditory cue. To test this, we injected a Cre-dependent excitatory chemogenetic virus (AAV8-hSyn-DIO-hM3Dq-mCherry) into the BA of GADCre(+) mice (Fig. 4*A*). As expected, the application of CNO increased the excitability (reduced the rheobase) of hM3Dq-expressing BA GABA neurons (Fig. 4*B*,*C*).

To assess the behavioral impact of this manipulation, we used a modified fear conditioning protocol in which subjects were given three presentations of an auditory cue/CS, without footshock/US (3 CS/0 US; Tipps et al., 2014). BA GABA neuron stimulation during training yielded increased freezing to the third CS presentation (CS3), relative to controls (Fig. 4*D*). Significantly enhanced freezing was also seen in GADCre(+)/hM3Dq subjects during the subsequent context and cue recall tests, conducted in the absence of CNO (Fig. 4*E***,***F*). Importantly, no group differences in freezing behavior were observed before the first CS presentation during training (Fig. 4*C*), or in response to the altered context during the cue recall test (Fig. 4*F*). Further, GADCre(+)/hM3Dq mice froze significantly more during the CS presentations during the cue test compared to the non-CS periods, whereas GADCre(−)/hM3Dq subjects did not. These findings suggest that BA GABA neuron stimulation does not elicit a general increase in freezing behavior, but rather, promotes the formation of an associative response to an otherwise neutral auditory cue and associated context.

### Chemogenetic stimulation of BA GABA neurons inhibits BA pyramidal neurons

**Figure 5.**
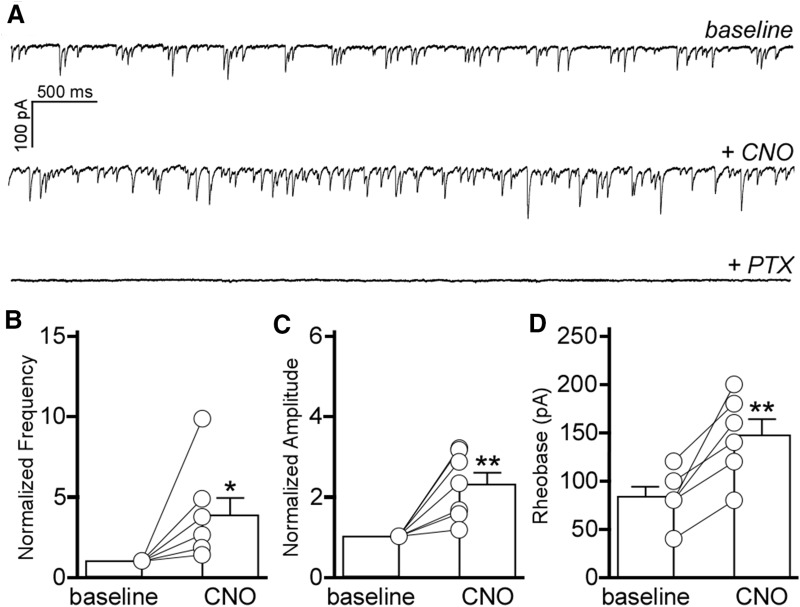
BA GABA neuron stimulation inhibits BA pyramidal neurons. ***A***, mIPSCs in BA pyramidal neurons from GADCre(+)/hM3Dq mice (*V*_hold_ = −70 mV). Traces show mIPSCs before (baseline) and after application of CNO (10 μm), and mIPSC sensitivity to the GABA_A_R antagonist PTX (100 μm). ***B***, Impact of CNO/hM3Dq-mediated activation of BA GABA neurons on mIPSC frequency in BA pyramidal neurons (*t*_(6)_=2.6, *p = 0.400). Post-CNO measures were normalized to baseline for each recording because of significant cell-to-cell variability in mIPSC activity (*n* = 7 experiments). ***C***, Impact of CNO/hM3Dq-mediated activation of BA GABA neurons on mIPSC amplitude in BA pyramidal neurons (*t*_(6)_=4.1, **p = 0.006); post-CNO measures were normalized to baseline values for each recording (*n* = 7 experiments). ***D***, Impact of CNO/hM3Dq-mediated activation of BA GABA neurons on rheobase in BA pyramidal neurons, at baseline and after CNO application (*t*_(5)_=4.8, **p = 0.005; *n* = 6 experiments).

Our data demonstrate that BA GABA neuron activity is necessary for fear memory acquisition (Fig. 3*D*) and sufficient to produce a long-term association between an auditory cue and associated context (Fig. 4*D–F*). GABA neurons in the BLA complex are thought to primarily be interneurons that regulate the activity of local pyramidal neurons (Woodruff and Sah, 2007; Spampanato et al., 2011; Veres et al., 2014, 2017; Vereczki et al., 2016). As such, the hM3Dq-mediated stimulation of BA GABA neurons should result in BA pyramidal neuron inhibition. To test this prediction, we measured mIPSCs in BA pyramidal neurons, before and after chemogenetic stimulation of BA GABA neurons. In slices from mice expressing hM3Dq in BA GABA neurons, bath application of CNO increased the frequency and amplitude of GABA_A_R-mediated mIPSCs in BA pyramidal neurons (Fig. 5*A–C*). Additionally, BA pyramidal neuron excitability, as assessed by rheobase, was decreased by the hM3Dq-mediated stimulation of BA GABA neurons (Fig. 5*D*). Thus, BA GABA neuron stimulation increases inhibitory input to BA pyramidal neurons, and suppresses BA pyramidal neuron excitability.

### Chemogenetic inhibition of BA pyramidal neurons promotes fear learning

**Figure 6.**
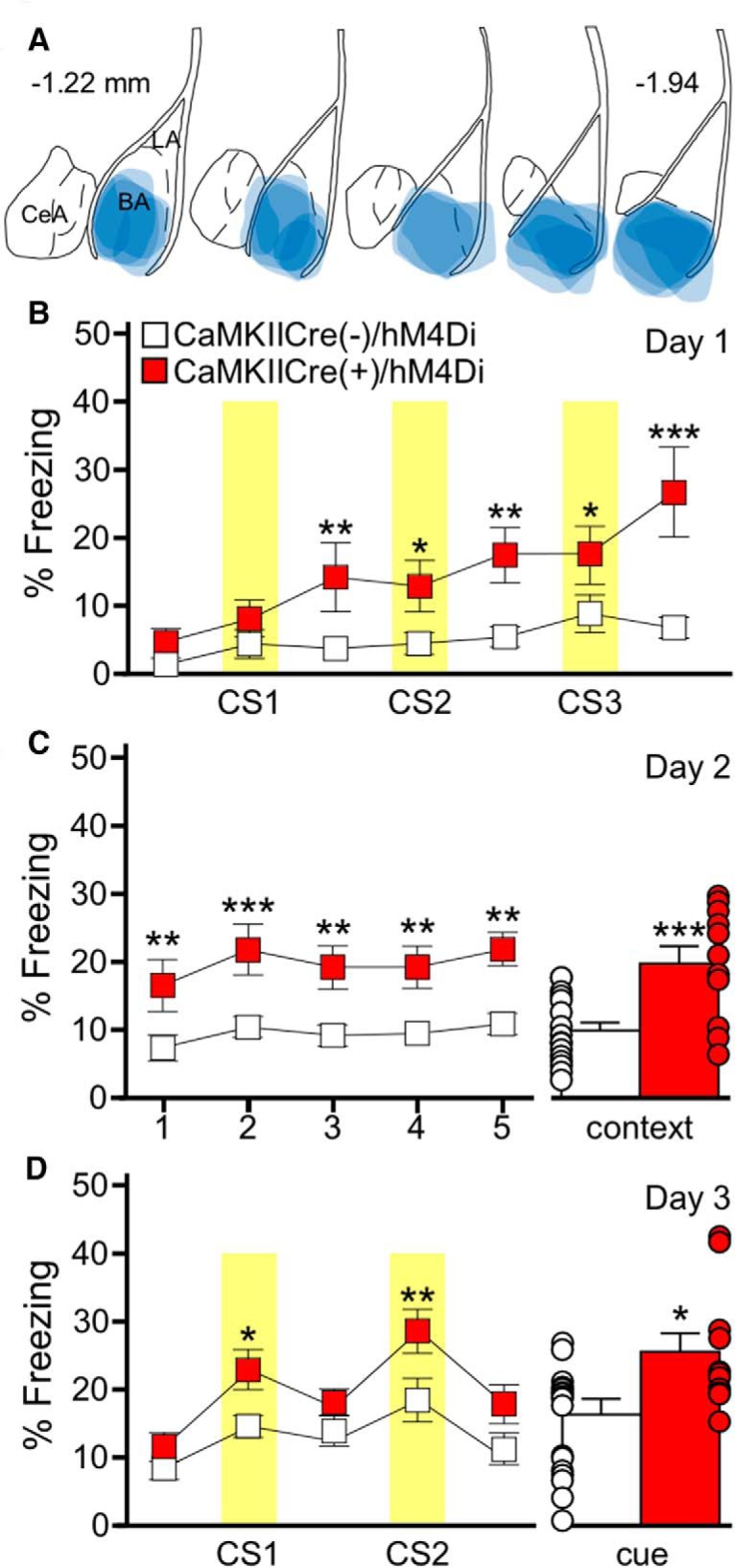
BA pyramidal neuron inhibition generates an association between a behavior and an auditory cue. ***A***, Schematic summarizing the distribution of hM4Di-mCherry fluorescence in the BA (coronal view, spanning –1.22mm to –1.94mm posterior from bregma) of the CaMKIICre(+)/hM4Di mice evaluated in ***B***–***D***. ***B***, Training with the 3 CS/0 US protocol in CaMKIICre(+)/hM4Di (red) and CaMKIICre(−)/hM4Di (white) mice. CNO (2 mg/kg, i.p.) was given 30 min before training. Yellow bars represent the CS presentations. There was a significant main effect of genotype (*F*_(1,162)_=14.1, *p* < 0.001; post-CS1: **p = 0.006, CS2: *p = 0.025, post-CS2: **p = 0.002, CS3: *p = 0.019, post-CS3: ***p < 0.001). ***C***, Freezing during the context recall test. The plot on the left shows freezing during each 1 min bin of the 5 min test. There was main effect of genotype (*F*_(1,104)_=16.1, *p* < 0.001; bin 1: **p = 0.007, bin 2: ***p < 0.001, bin 3: **p = 0.004, bin 4: **p = 0.004, bin 5: **p = 0.001). Error bars on the right show mean percentage freezing for the full 5 min context test (*t*_(29)_= −4.2, ***p < 0.001). ***D***, Freezing during the cue recall test. The plot on the left shows freezing during each 3 min portion of the test, with yellow bars representing the CS presentations. There was a significant effect of genotype (*F*_(1,108)_=5.2, *p* = 0.03; CS1: *p = 0.02, CS2: **p = 0.005). CaMKIICre(+)/hM4Di mice (*t*_(24)_= −3.3, *p* = 0.004), but not CaMKIICre(−)/hM4Di mice (*t*_(34)_=−1.8, *p* = 0.080) froze significantly more during the CS presentations compared to the non-CS periods. The bars on the right show mean percentage freezing during the combined CS presentations (*t*_(29)_=−2.2, *p = 0.039). Error bars in ***B–D*** represent the mean ± SEM, with dots next to each bar denoting the individual data points (*n* = 6/7–10/8 males/females per group).

Given that BA GABA neuron stimulation is sufficient to generate a long-term associative response to a neutral auditory cue, and that BA GABA neuron stimulation inhibits BA pyramidal neurons, we next asked whether the hM4Di-mediated inhibition of BA pyramidal neurons during training could also induce the formation of a long-term behavioral response to a neutral auditory cue. We again used CaMKIICre(+) mice and an AAV8-hSyn-DIO-hM4Di-mCherry virus to express hM4Di in BA pyramidal neurons (Fig. 6*A**)*, and administered CNO to viral-treated CaMKIICre(+) and CaMKIICre(−) subjects 30 min before training in the 3 CS/0 US paradigm. Similar to the outcome of the BA GABA neuron stimulation experiments, BA pyramidal neuron inhibition promoted freezing to the second and third CS presentations during training, but had no effect on freezing before the first CS presentation (Fig. 6*B*). Moreover, BA pyramidal neuron inhibition during training resulted in the formation of a long-term associative response, as revealed by increased freezing relative to control subjects in both the context and cue recall tests (Fig. 6*C***,***D*). Importantly, no group differences were observed on initial introduction of the subjects to the altered context during the cue test (Fig. 6*D*). Further, CaMKIICre(+)/hM4Di mice, but not CaMKIICre(−)/hM4Di mice, froze significantly more during the CS presentations during the cue test compared to the non-CS periods. These findings suggest that chemogenetic inhibition of BA pyramidal neurons does not elicit a general increase in freezing behavior, but rather, supports the formation of an associative response to an otherwise neutral auditory cue and associated context.

**Figure 7.**
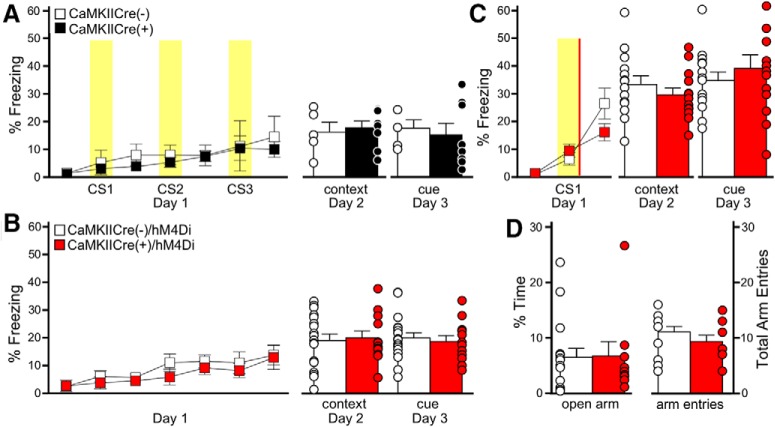
Induction of memory formation by BA pyramidal neuron inhibition is not because of genotype differences, nonspecific freezing behavior, or anxiety changes. ***A***, Genotype difference test. Training with the 3 CS/0 US protocol in untreated CaMKIICre(+) (black) and CaMKIICre(−) (white) mice is shown on the left. Yellow bars represent the CS presentations. There was no effect of genotype (*F*_(1,66)_=0.37, *p* = 0.55). Freezing during context (*t*_(11)_=−0.4, *p* = 0.73) and cue (*t*_(11)_=0.4, *p* = 0.69) recall tests for untreated CaMKIICre(+) and CaMKIICre(−) littermates, following conditioning with the 3 CS/0 US protocol, is plotted on the right. Sex effects were not analyzed for this study (*n* = 5–8/genotype). ***B***, Nonspecific freezing behavior test. Training with the 0 CS/0 US protocol in CaMKIICre(+)/hM4Di (red) and CaMKIICre(−)/hM4Di (white) mice is shown on the left. CNO (2 mg/kg, i.p.) was given 30 min before training. No CS or US presentations were given. The plot on the left show freezing during training. No genotype differences were seen during training (*F*_(1,168)_=0.5, *p* = 0.49). The plots on the right show freezing during context (*t*_(28)_=−0.0, *p* = 0.98) and cue (*t*_(28)_=−0.5, *p* = 0.65) recall tests (*n* = 6/6–8/10 males/females per group). No genotype differences were detected in average motion (*F*_(1,140)_=0.9, *p* = 0.35) or maximum motion (*F*_(1,140)_=0.95, *p* = 0.34) indices (data not shown). ***C***, One CS/US protocol test. Freezing following training with a weak (1 CS/1 US) fear conditioning protocol in CaMKIICre(+)/hM4Di (red) and CaMKIICre(−)/hM4Di (white) mice is shown on the left. CNO (2 mg/kg, i.p.) was given 30 min before training. The yellow bar represents the CS presentation and the red bar represents the US presentation. There was no effect of genotype (*F*_(1,50)_=0.83, *p* = 0.37). The plots on the right show freezing during context (*t*_(25)_=1.0, *p* = 0.35) and cue (*t*_(25)_=−0.8, *p* = 0.46) recall tests (*n* = 7/6–7/7 males/females per group). ***D***, Anxiety test. Percentage of time spent in the open arms (***D***; *t*_(23)_=0.1, *p* = 0.94) by CaMKIICre(+)/hM4Di (red) and CaMKIICre(−)/hM4Di (white) mice and total number of arm entries (***E***; *t*_(23)_=1.1, *p* = 0.28). All subjects received CNO (2 mg/kg, i.p.) 30 min before EPM testing (*n* = 5/5–8/7 males/females group). Bars represent the mean ± SEM, with dots next to each bar denoting the individual data points.

Although these data suggest that BA pyramidal neuron inhibition is a key contributor to associative memory formation, we designed a series of control experiments to test alternative explanations. First, although our study design ensured that all animals received the same viral construct and CNO treatment, a baseline difference in fear learning between CaMKIICre(−) and CaMKIICre(+) littermates could explain our observations. There was no difference in freezing behavior, however, during training or recall tests for untreated CaMKIICre(+) and CaMKIICre(−) littermates trained with the 3 CS/0 US protocol, in the absence of CNO (Fig. 7*A*). Second, although freezing levels were similar across groups before the initial CS presentation during training, and on initial exposure to the altered context during the cue test, we conducted additional tests to determine if chemogenetic inhibition of BA pyramidal neurons resulted in locomotor impairments and/or nonspecific freezing behavior. Chemogenetic inhibition of BA pyramidal neurons had no impact on freezing behavior or the average motion index or maximum motion measures during training using a 0 CS/0 US paradigm (context exposure only; Fig. 7*B*). Freezing behavior in the subsequent recall tests was also unaffected *(*Fig. 7*B*). In addition to demonstrating that chemogenetic inhibition of BA pyramidal neurons does not evoke a nonspecific increase in freezing behavior (or a nonspecific decrease in locomotion), these data also show that the learned association generated by BA pyramidal neuron inhibition requires a discrete CS presentation.

The dependence of the CNO/hM4Di-induced fear memory on CS presentation raised the possibility that BA pyramidal neuron inhibition during training may simply strengthen an otherwise weak association between the US and auditory CS. To test this prospect, we used a weak conditioning protocol involving a single CS–US pairing (1 CS/1 US), which would allow us to detect an increase in freezing behavior that might be obscured in more robust standard (3 CS/3 US) conditioning protocol. BA pyramidal neuron inhibition during conditioning in the 1 CS/1 US protocol did not alter freezing during training or enhance context or cue-induced freezing in recall tests (Fig. 7*C*). Thus, although BA pyramidal neuron inhibition may induce memory formation, it does not determine the strength of the association when a US is presented.

Finally, as anxiety can impact performance in fear conditioning tests (Izquierdo et al., 2016), we asked whether BA pyramidal neuron inhibition altered anxiety-related behavior, using the EPM test. BA pyramidal neuron inhibition did not alter time spent in the open arms of the EPM or number of total (open + closed) arm entries (Fig. 7*D*), indicating that BA pyramidal neuron inhibition does not impact anxiety-related behavior or general motor activity. Collectively, these data also suggest that inhibition of BA pyramidal neurons does not promote a subjective sense of fear, which would be expected to generate increased freezing in both the 0 CS/0 US and 1 CS/1 US conditions, and potentially decrease the time spent in the open arms of the EPM.

### Chemogenetic induction of fear learning requires GIRK channel activation in BA pyramidal neurons

Chemogenetic tools regulate neuronal excitability by using endogenous G protein-dependent signal transduction pathways. For example, hM4Di is thought to inhibit neurons by activating inhibitory (G_i/o_) G-proteins (Armbruster et al., 2007). G-protein-gated inwardly rectifying K^+^ (GIRK) channels mediate G_i/o_-dependent signaling in many neuron populations (Lüscher and Slesinger, 2010; Lüjan et al., 2014), including BA pyramidal neurons (Marron Fernandez de Velasco et al., 2017), and they have been implicated in the inhibitory effect of hM4Di (Armbruster et al., 2007). Thus, as a final test for the specificity of our chemogenetic manipulation on associative learning, we asked whether the hM4Di-dependent inhibition of BA pyramidal neurons could be blocked by GIRK channel ablation.

**Figure 8.**
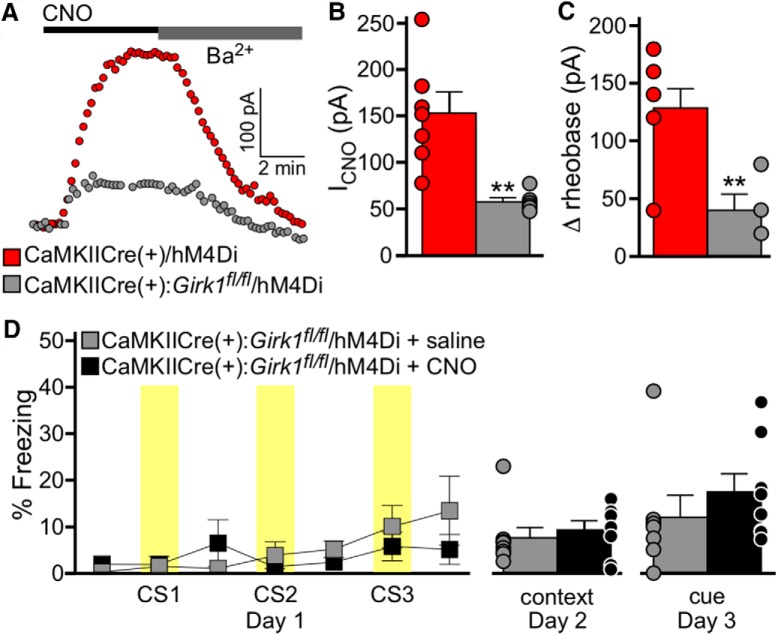
Chemogenetic induction of associative learning requires GIRK channel activation. ***A***, Currents evoked by CNO (10 μm) in BA pyramidal neurons from CaMKIICre(+)/hM4Di (*Girk1^+/+^*; red) and CaMKIICre(+):*Girk1^fl/fl^/*hM4Di (*Girk1^–/–^*; gray) mice. Currents were reversed by bath application of 0.3 mm Ba^2+^. ***B***, Summary of CNO-induced currents in hM4Di-positive BA pyramidal neurons from CaMKIICre(+)/hM4Di (*Girk1^+/+^*; red) and CaMKIICre(+):*Girk1^fl/^* ^fl^/hM4Di (*Girk1^–/–^*; gray) mice (*t*_(11)_=3.9, **p = 0.003; *n* = 6–7/group). ***C***, CNO-induced change in rheobase (Δrheobase) in hM4Di-positive BA pyramidal neurons from CaMKIICre(+)/hM4Di (*Girk1^+/+^*; red) and CaMKIICre(+):*Girk1^fl/fl^*/hM4Di (*Girk1^–/–^*; gray) mice (*t*_(9)_=3.6, **p = 0.006; *n* = 6–7/group). ***D***, Saline (gray) or CNO (2 mg/kg, i.p.; black) was administered to CaMKIICre(+):*Girk1^fl/fl^*/hM4Di mice, 30 min before training in the 3 CS/0 US protocol. Freezing during training is plotted on the left. Yellow bars represent the CS presentations. There was no effect of genotype (*F*_(1,84)_=0.3, *p* = 0.58). Freezing during context (*t*_(14)_=0.6, *p* = 0.57) and cue (*t*_(13)_=0.9, *p* = 0.38) recall tests is plotted on the right (*n* = 8 mice/group). Bars represent the mean ± SEM, with dots next to each bar denoting the individual data points. Sex effects were not analyzed for this study.

We used a neuron-specific *Girk1^–/–^* mouse line [CaMKIICre(+):*Girk1^fl/fl^* mice] that lacks GIRK channel activity in pyramidal neurons in multiple brain regions, including the BA (Marron Fernandez de Velasco et al., 2017). In hM4Di-expressing BA pyramidal neurons from CaMKIICre(+) (control/Girk^+/+^) mice, CNO evoked an outward/inhibitory current that was reversed by 0.3 mm extracellular Ba^2+^, which blocks inwardly rectifying channels, including GIRK channels (Fig. 8*A*). In hM4Di-expressing BA pyramidal neurons from CaMKIICre(+):*Girk1^fl/fl^* mice, CNO-induced currents were significantly smaller (Fig. 8*A***,***B*), and the inhibitory influence of CNO on BA pyramidal neuron excitability (shown as change in rheobase) was blunted (Fig. 8*C*). Thus, GIRK channel activation mediates most of the inhibitory effect of hM4Di activation on BA pyramidal neurons.

To test whether GIRK channel activation is also required for the chemogenetic induction of associative learning, we evaluated CaMKIICre(+):*Girk1^fl/fl^* mice in the 3 CS/0 US conditioning protocol, 4 weeks after intra-BA infusion of AAV8-hSyn-DIO-hM4Di-mCherry virus. Mice were randomly assigned to receive saline or CNO 30 min before training. The levels of freezing observed during training, and in subsequent context and cue recall tests, did not differ between saline- and CNO-treated subjects (Fig. 8*D*). Thus, the loss of GIRK channels, a primary mediator of hM4Di influence in BA pyramidal neurons, is sufficient to block the generation of an associative behavioral response via chemogenetic inhibition of BA pyramidal neurons.

## Discussion

Inhibitory interneurons regulate the activity of local excitatory principal neurons in many brain regions. These interneurons are critical for shaping network activity and exhibit experience-induced plasticity, suggesting that changes in inhibitory signaling may underlie long-term learning and behavioral changes (Lucas and Clem, 2018). Within the field of learning and memory, the impact of inhibitory interneuron activity is well illustrated by the disinhibition model of associative fear memory formation (Letzkus et al., 2015). In this model, the combined effect of exposure to a CS and US during training results in an overall decrease in GABAergic input to glutamatergic projection neurons, leading to an increase in projection neuron excitability. Examples of disinhibition-based signaling can be found in several brain regions, including the cortex (Letzkus et al., 2011) and BLA complex (Wolff et al., 2014).

Available data support the contention that excitation of LA pyramidal neurons is a critical step in auditory fear conditioning. For example, optogenetic stimulation of LA pyramidal neurons in mice can generate or enhance fear memory formation (Johansen et al., 2010; Yiu et al., 2014), whereas optogenetic (Johansen et al., 2014) or chemogenetic (Fig. 2) inhibition of LA pyramidal neurons impairs fear learning. In addition, the excitability of LA pyramidal neurons correlates positively with inclusion of those neurons in the subsequent fear memory trace (Zhou et al., 2009; Kim et al., 2014). Consistent with a key role for pyramidal neuron excitation in fear memory formation, the combined effect of exposure to a CS and US during fear conditioning results in an overall decrease in GABAergic input to pyramidal neurons of the BLA complex, resulting in pyramidal neuron disinhibition (Wolff et al., 2014). Indeed, manipulations of the parvalbumin-expressing subtype of GABA neurons in the BLA complex have been shown to modulate the strength of associative memory formation in response to a footshock, with inhibition of parvalbumin-expressing neurons during the CS–US presentation increasing fear learning and stimulation of parvalbumin-expressing neurons impairing fear learning (Wolff et al., 2014).

While studies have consistently illustrated the role of LA pyramidal neuron excitation in auditory fear conditioning, most of the published lesion and pharmacological inhibition studies have not supported a role for the BA in the acquisition of cue fear memories (Nader et al., 2001; Anglada-Figueroa and Quirk, 2005; Calandreau et al., 2005; Onishi and Xavier, 2010; Akagi Jordão et al., 2015). However, given that lesions and pharmacologic inhibitors lack neuronal specificity and can have broad and nonspecific effects on neural circuits, we revisited the role of the BA in auditory fear learning using a neuron-specific chemogenetic approach. We found that activation of BA GABA neurons and inhibition of BA pyramidal neurons are critical steps in the formation of associative fear memories. Given that optogenetic stimulation of LA principal neurons (Johansen et al., 2014), and chemogenetic inhibition of BA pyramidal neurons (Fig. 6), can both promote the formation of learned response to a neutral auditory cue, our data suggest the intriguing prospect that the fear conditioning-induced disinhibition of LA pyramidal neurons triggers the stimulation of BA GABA neurons and feedforward inhibition of BA pyramidal neurons.

This conceptual framework can reconcile apparently contradictory prior observations related to the impact of lesions, as well as pharmacologic and genetic interventions, on auditory fear conditioning. For example, the lack of impact of pharmacological inactivation of the BA during auditory fear conditioning was interpreted as evidence that the BA is not required for fear learning (Calandreau et al., 2005). Given that chemogenetic inhibition of BA pyramidal neurons also did not preclude fear learning (Fig. 2), we speculate that the potentially detrimental effect on fear learning of inhibiting BA GABA neurons using a broad pharmacological approach might be offset by the direct inhibition of BA pyramidal neurons. Similarly, a system in which LA pyramidal neuron inhibition significantly impairs long-term memory formation, whereas BA pyramidal neuron inhibition does not, could explain why some manipulations of pyramidal neurons across the entire BLA complex during training resulted in significant impairments observed during recall testing (Wolff et al., 2014), whereas others did not (Namburi et al., 2015). Our results show that inhibitory manipulations of pyramidal neurons within the BLA complex that bias toward the LA would be expected to impair fear memory formation, whereas the same manipulation primarily targeted within the BA would not. A more extensive analysis of these manipulations in the LA will be needed to confirm this hypothesis, however. The work presented here focuses on the BA, primarily based on our initial finding that pyramidal inhibition in the LA produced the anticipated impairment in fear memory formation, whereas BA pyramidal neuron inhibition did not. Although our investigation of this interesting distinction yielded novel results, the application of our approach to the LA may also yield surprising effects, and will be an important direction for future work.

Our data suggest an interesting extension of the circuitry and signaling mechanisms implicated in auditory fear learning; however, limitations associated with the approaches used in this study are worth noting. First, although the CaMKIICre and GADCre transgenic mouse lines used in this study facilitated the manipulation of distinct neuron populations, pyramidal and GABA neurons in the BLA complex are diverse. The GADCre line, for example, drives expression in all major GABA neuron subtypes (Taniguchi et al., 2011), including the PV and somatostatin subtypes that exert opposing influence on LA pyramidal neuron activity (Lovett-Barron et al., 2014; Wolff et al., 2014; Lucas and Clem, 2018). Thus, extending our efforts to identify the relevant subpopulation(s) of inhibitory interneurons that are essential for auditory fear conditioning will be informative. Similarly, the afferent and efferent connections of pyramidal neurons in the BLA complex are diverse, and these distinctions have significant implications for both conditioned fear and anxiety (Janak and Tye, 2015; Beyeler et al., 2016, 2018; Burgos-Robles et al., 2017). Thus, it will be interesting to use projection-specific chemogenetic manipulations of discrete BA microcircuits to understand which projection(s) is/are most relevant to the facilitation of associative learning by inhibitory signaling reported in this study.

We also note that the time course of the chemogenetic manipulations used in this study encompassed most of the acquisition and consolidation periods (Roth, 2016). Although this design allowed us to probe the role of inhibitory signaling without an a priori assumption regarding when such signaling might be relevant, future work using a more temporally discrete approach will be needed to identify critical time points within this period for the inhibitory mechanisms identified in this study. A more temporally restricted approach will also allow for additional control measures, such as the inclusion of a CS presented in the absence of neuronal inhibition, to further validate the specificity of our reported effects. On a related front, although our work focuses on the acquisition of new fear memories, the BA has been implicated in the recall and extinction of established fear memories (Herry et al., 2008; Amano et al., 2011), and the implications of our findings to the role played by this brain region in other aspects of fear learning is unclear.

It is also important to note that pyramidal neurons in the BLA complex also receive excitatory input during CS and US presentations (Letzkus et al., 2015). Our demonstration that BA pyramidal neuron inhibition promotes the association between an auditory cue and a behavioral response does not rule out a role for BA pyramidal neuron excitation in associative learning as well. Indeed, the inability of BA pyramidal neuron inhibition to generate a long-term memory in the absence of a discrete CS, and the lack of impact of BA pyramidal neuron inhibition in a weak fear conditioning paradigm (Fig. 7), suggest that other signals shape the resulting fear memory.

Together, our findings demonstrate that inhibitory signaling in the amygdala plays a more diverse and nuanced role in associative learning than originally thought. In combination with previous studies, our work shows that the cellular mechanisms underlying fear learning differ in the LA and BA, with pyramidal neuron excitation promoting memory formation in the former, and pyramidal neuron inhibition serving this role in the latter. It will be important to investigate more extensively the potential role of inhibitory signaling in normal associative learning processes, and in diseases in which these processes are disrupted.

## Synthesis

Reviewing Editor: Karen Szumlinski, University of California at Santa Barbara Decisions are customarily a result of the Reviewing Editor and the peer reviewers coming together and discussing their recommendations until a consensus is reached. When revisions are invited, a fact-based synthesis statement explaining their decision and outlining what is needed to prepare a revision will be listed below. The following reviewer(s) agreed to reveal their identity: Francisco Sotres-Bayon. Note: If this manuscript was transferred from JNeurosci and a decision was made to accept the manuscript without peer review, a brief statement to this effect will instead be what is listed below.

After consultation with the two original reviewers of your transferred article, we have reached the decision of “Revise-Editorial Review Only”. This decision was based on the acknowledgement that the authors have toned down their conclusions and provided appropriate discussion of the pitfalls and short-comings of their study. While both reviewers would like to see the present set of results followed up in future experiments, they are understanding of the labortory's situation and are willing to make concessions about the inclusion of more data. This being said, both reviewers have remaining concerns that linger from the previous round of comments that I will review for inclusion what we hope to be the final revision to this report. Please find the specific comments of the reviewers below:

Reviewer 1:

The authors have made some changes but are not fully responsive to prior critiques.

1. The authors continue to insist that there are no acquisition effects in Figure 2J by arguing that the inhibition of BA pyramidal neurons had no effect on the acquisition of *long-term* memories (lines 261-262). I do not understand this response. In truth, there is no effect on the retention but there is clearly an effect on acquisition. ‘Acquisition’, as used in standard learning and memory work, refers to the initial learning period, which is the left-side panel of Figure 2J. The authors show asterisks for CS3 and the period immediately after, meaning there is an effect on acquisition -- even if this difference did not alter the retention. I would suggest the authors correct this issue in the manuscript.

2. I appreciate the authors' inclusion of additional points in the Discussion, particularly regarding gating. However, the way authors use ‘gating’ and the way Luthi and others use the term are different. For Luthi (e.g., the Wolff 2014 paper the authors repeatedly cite), gating plays a permissive or enabling role for the synaptic plasticity underlying the memory formation. Thus, gating mechanisms can be manipulated to either enhance or impair the memory formation. Wolff et al. (2014) use CS-US pairings throughout the experiments (in contrast to the effects the authors of this manuscript find without a US) and demonstrate that PV neuron manipulations can enhance and impair the memory. Luthi's group argues that the PV neurons play a role in gating the CS-US associations.

In contrast, the authors of the present manuscript demonstrate that activation of BA neurons in the absence of a US can create a memory, yet such activation has no effect on a single CS-US pairing. At minimum, if this ‘gating’ were like Luthi's gating, such activation would enhance the memory formation of the single CS-US pairing. Considering the novelty of the authors' findings, the authors do themselves (and their findings) a disservice by suggesting that their findings are of the same kind as Wolff et al. The authors' findings are different and consequently rather interesting. I would suggest the authors refrain from referring to their findings as ‘gating’ or, at minimum, suggest that, if this is gating, it is different from what Wolff found in their work.

Reviewer 2:

This timely study with relevant findings extend an important literature on the study of associative learning mechanisms by highlighting a novel role for inhibitory signaling in the amygdala.

In this revised version, the authors addressed the concerns raised by making relevant changes to the text of the manuscript and figures. This changes included toning down the interpretations of their findings and thereby changing the title. Since there is no direct evidence for gating mechanism I suggest the authors mention this concept to a minimum.

## Author Response

We would like to thank the reviewers and the editor for their helpful comments. We deeply appreciate the time the editor in particular has dedicated to the review process. Our response to the minor comments from the reviewers are given below in red. Likewise, the corresponding changes to the manuscript are highlighted in red in the main article file.

Reviewer 1:

The authors have made some changes but are not fully responsive to prior critiques.

1. The authors continue to insist that there are no acquisition effects in Figure 2J by arguing that the inhibition of BA pyramidal neurons had no effect on the acquisition of *long-term* memories (lines 261-262). I do not understand this response. In truth, there is no effect on the retention but there is clearly an effect on acquisition. ‘Acquisition’, as used in standard learning and memory work, refers to the initial learning period, which is the left-side panel of Figure 2J. The authors show asterisks for CS3 and the period immediately after, meaning there is an effect on acquisition -- even if this difference did not alter the retention. I would suggest the authors correct this issue in the manuscript.

While the acquisition period is a critical portion of the learning process, differences in this period do not necessarily reflect changes in learning. Auditory startle, footshock sensitivity, and many other factors can lead to significantly different freezing levels during acquisition that do not translate into changes in the resulting memory. As such, the learning field as a whole does not rely on differences during acquisition to define changes in learning/memory. Indeed, most papers do not even provide training data, because differences in these period are so hard to interpret. At no point in our paper do we use training data alone to define our effects. Our decision to show this data was based on our desire for full transparency and to provide proof that our DREADD manipulation was not drastically altering baseline locomotion or tone responsivity.

We maintain that without a significant difference in the recall tests, we cannot claim there was an effect of BA pyramidal neuron inhibition on learning, as defined through the traditional use of recall tests. However, we see how our previous wording of this section led to confusion regarding our claims. It is not our intention to obscure the presented data. We have re-worded this section based on the reviewer's suggestion: page 11, lines 60-64.

2. I appreciate the authors' inclusion of additional points in the Discussion, particularly regarding gating. However, the way authors use ‘gating’ and the way Luthi and others use the term are different. For Luthi (e.g., the Wolff 2014 paper the authors repeatedly cite), gating plays a permissive or enabling role for the synaptic plasticity underlying the memory formation. Thus, gating mechanisms can be manipulated to either enhance or impair the memory formation. Wolff et al. (2014) use CS-US pairings throughout the experiments (in contrast to the effects the authors of this manuscript find without a US) and demonstrate that PV neuron manipulations can enhance and impair the memory. Luthi's group argues that the PV neurons play a role in gating the CS-US associations.

In contrast, the authors of the present manuscript demonstrate that activation of BA neurons in the absence of a US can create a memory, yet such activation has no effect on a single CS-US pairing. At minimum, if this ‘gating’ were like Luthi's gating, such activation would enhance the memory formation of the single CS-US pairing. Considering the novelty of the authors' findings, the authors do themselves (and their findings) a disservice by suggesting that their findings are of the same kind as Wolff et al. The authors' findings are different and consequently rather interesting. I would suggest the authors refrain from referring to their findings as ‘gating’ or, at minimum, suggest that, if this is gating, it is different from what Wolff found in their work.

Please see full response below.

Reviewer 2:

This timely study with relevant findings extend an important literature on the study of associative learning mechanisms by highlighting a novel role for inhibitory signaling in the amygdala.

In this revised version, the authors addressed the concerns raised by making relevant changes to the text of the manuscript and figures. This changes included toning down the interpretations of their findings and thereby changing the title. Since there is no direct evidence for gating mechanism I suggest the authors mention this concept to a minimum.

As both reviewers have expressed justifiable concerns regarding the use of the term ‘gating’ to describe our effects, we have removed this section from the manuscript. We refer to our effects as ‘promoting’ fear memory formation/learning throughout: page 21, lines 87 and 95. Further, we have limited our discussion of the work by Luthi et al, which is now included only as further evidence supporting the role of pyramidal neuron excitation in the LA during fear memory formation: text removed from the Discussion section: page 21.

## References

[B1] Akagi Jordão EM, Onishi BK, Xavier GF (2015) Pre-training reversible inactivation of the basal amygdala (BA) disrupts contextual, but not auditory, fear conditioning, in rats. PloS One 10:e0125489. 10.1371/journal.pone.0125489 25928357 PMC4415935

[B2] Amano T, Duvarci S, Popa D, Pare D (2011) The fear circuit revisited: contributions of the basal amygdala nuclei to conditioned fear. J Neurosci 31:15481–15489. 10.1523/JNEUROSCI.3410-11.2011 22031894 PMC3221940

[B3] Amorapanth P, LeDoux JE, Nader K (2000) Different lateral amygdala outputs mediate reactions and actions elicited by a fear-arousing stimulus. Nat Neurosci 3:74–79. 10.1038/71145 10607398

[B4] Anglada-Figueroa D, Quirk GJ (2005) Lesions of the basal amygdala block expression of conditioned fear but not extinction. J Neurosci 25:9680–9685. 10.1523/JNEUROSCI.2600-05.2005 16237172 PMC6725741

[B5] Armbruster BN, Li X, Pausch MH, Herlitze S, Roth BL (2007) Evolving the lock to fit the key to create a family of G protein-coupled receptors potently activated by an inert ligand. Proc Natl Acad Sci U S A 104:5163–5168. 10.1073/pnas.0700293104 17360345 PMC1829280

[B6] Arora D, Haluk DM, Kourrich S, Pravetoni M, Fernández-Alacid L, Nicolau JC, Luján R, Wickman K (2010) Altered neurotransmission in the mesolimbic reward system of *Girk–/–* mice. J Neurochem 114:1487–1497. 10.1111/j.1471-4159.2010.06864.x 20557431 PMC2941778

[B7] Beyeler A, Chang CJ, Silvestre M, Lévêque C, Namburi P, Wildes CP, Tye KM (2018) Organization of valence-encoding and projection-defined neurons in the basolateral amygdala. Cell Rep 22:905–918. 10.1016/j.celrep.2017.12.097 29386133 PMC5891824

[B8] Beyeler A, Namburi P, Glober GF, Simonnet C, Calhoon GG, Conyers GF, Luck R, Wildes CP, Tye KM (2016) Divergent routing of positive and negative information from the amygdala during memory retrieval. Neuron 90:348–361. 10.1016/j.neuron.2016.03.004 27041499 PMC4854303

[B9] Bocchio M, Fucsina G, Oikonomidis L, McHugh SB, Bannerman DM, Sharp T, Capogna M (2015) Increased serotonin transporter expression reduces fear and recruitment of parvalbumin interneurons of the amygdala. Neuropsychopharmacology 40:3015–3026. 10.1038/npp.2015.157 26052039 PMC4864439

[B10] Bowers ME, Ressler KJ (2015) An overview of translationally informed treatments for posttraumatic stress disorder: animal models of Pavlovian fear conditioning to human clinical trials. Biol Psychiatry 78:E15–E27. 10.1016/j.biopsych.2015.06.008 26238379 PMC4527085

[B11] Burgos-Robles A, Kimchi EY, Izadmehr EM, Porzenheim MJ, Ramos-Guasp WA, Nieh EH, Felix-Ortiz AC, Namburi P, Leppla CA, Presbrey KN, Anandalingam KK, Pagan-Rivera PA, Anahtar M, Beyeler A, Tye KM (2017) Amygdala inputs to prefrontal cortex guide behavior amid conflicting cues of reward and punishment. Nat Neurosci 20:824–835. 10.1038/nn.4553 28436980 PMC5448704

[B12] Calandreau L, Desmedt A, Decorte L, Jaffard R (2005) A different recruitment of the lateral and basolateral amygdala promotes contextual or elemental conditioned association in Pavlovian fear conditioning. Learn Mem 12:383–388. 10.1101/lm.92305 16027178 PMC1183256

[B13] Duvarci S, Pare D (2014) Amygdala microcircuits controlling learned fear. Neuron 82:966–980. 10.1016/j.neuron.2014.04.042 24908482 PMC4103014

[B14] Fanselow MS, LeDoux JE (1999) Why we think plasticity underlying Pavlovian fear conditioning occurs in the basolateral amygdala. Neuron 23:229–232. 10399930 10.1016/s0896-6273(00)80775-8

[B15] Fanselow MS, Poulos AM (2005) The neuroscience of mammalian associative learning. Annu Rev Psychol 56:207–234. 10.1146/annurev.psych.56.091103.070213 15709934

[B16] Goosens KA, Maren S (2001) Contextual and auditory fear conditioning are mediated by the lateral, basal, and central amygdaloid nuclei in rats. Learn Mem 8:148–155. 10.1101/lm.37601 11390634 PMC311374

[B17] Hearing M, Kotecki L, Marron Fernandez de Velasco E, Fajardo-Serrano A, Chung HJ, Luján R, Wickman K (2013) Repeated cocaine weakens GABA(B)-Girk signaling in layer 5/6 pyramidal neurons in the prelimbic cortex. Neuron 80:159–170. 10.1016/j.neuron.2013.07.019 24094109 PMC3793643

[B18] Herry C, Johansen JP (2014) Encoding of fear learning and memory in distributed neuronal circuits. Nat Neurosci 17:1644–1654. 10.1038/nn.3869 25413091

[B19] Herry C, Ciocchi S, Senn V, Demmou L, Müller C, Lüthi A (2008) Switching on and off fear by distinct neuronal circuits. Nature 454:600–606. 10.1038/nature07166 18615015

[B20] Izquierdo I, Furini CR, Myskiw JC (2016) Fear memory. Physiol Rev 96:695–750. 10.1152/physrev.00018.2015 26983799

[B21] Janak PH, Tye KM (2015) From circuits to behaviour in the amygdala. Nature 517:284–292. 10.1038/nature14188 25592533 PMC4565157

[B22] Johansen JP, Hamanaka H, Monfils MH, Behnia R, Deisseroth K, Blair HT, LeDoux JE (2010) Optical activation of lateral amygdala pyramidal cells instructs associative fear learning. Proc Natl Acad Sci U S A 107:12692–12697. 10.1073/pnas.1002418107 20615999 PMC2906568

[B23] Johansen JP, Cain CK, Ostroff LE, LeDoux JE (2011) Molecular mechanisms of fear learning and memory. Cell 147:509–524. 10.1016/j.cell.2011.10.009 22036561 PMC3215943

[B24] Johansen JP, Diaz-Mataix L, Hamanaka H, Ozawa T, Ycu E, Koivumaa J, Kumar A, Hou M, Deisseroth K, Boyden ES, LeDoux JE (2014) Hebbian and neuromodulatory mechanisms interact to trigger associative memory formation. Proc Natl Acad Sci U S A 111:E5584–E5592. 10.1073/pnas.1421304111 25489081 PMC4280619

[B25] Kim J, Kwon JT, Kim HS, Josselyn SA, Han JH (2014) Memory recall and modifications by activating neurons with elevated CREB. Nat Neurosci 17:65–72. 10.1038/nn.3592 24212670

[B26] Krettek JE, Price JL (1978) A description of the amygdaloid complex in the rat and cat with observations on intra-amygdaloid axonal connections. J Comp Neur 178:255–280. 10.1002/cne.901780205 627626

[B27] LeDoux JE (2000) Emotion circuits in the brain. Annu Rev Neurosci 23:155–184. 10.1146/annurev.neuro.23.1.155 10845062

[B28] LeDoux JE, Cicchetti P, Xagoraris A, Romanski LM (1990) The lateral amygdaloid nucleus: sensory interface of the amygdala in fear conditioning. J Neurosci 10:1062–1069. 2329367 10.1523/JNEUROSCI.10-04-01062.1990 PMC6570227

[B29] Letzkus JJ, Wolff SB, Lüthi A (2015) Disinhibition: a circuit mechanism for associative learning and memory. Neuron 88:264–276. 10.1016/j.neuron.2015.09.024 26494276

[B30] Letzkus JJ, Wolff SB, Meyer EM, Tovote P, Courtin J, Herry C, Lüthi A (2011) A disinhibitory microcircuit for associative fear learning in the auditory cortex. Nature 480:331–335. 10.1038/nature10674 22158104

[B31] Lovett-Barron M, Kaifosh P, Kheirbek MA, Danielson N, Zaremba JD, Reardon TR, Turi GF, Hen R, Zemelman BV, Losonczy A (2014) Dendritic inhibition in the hippocampus supports fear learning. Science 343:857–863. 10.1126/science.1247485 24558155 PMC4018419

[B32] Lucas EK, Clem RL (2018) GABAergic interneurons: the orchestra or the conductor in fear learning and memory? Brain Res Bull 141:13–19. 10.1016/j.brainresbull.2017.11.016 29197563 PMC6178932

[B33] Lüjan R, Marron Fernandez de Velasco E, Aguado C, Wickman K (2014) New insights into the therapeutic potential of Girk channels. Trends Neurosci 37:20–29. 10.1016/j.tins.2013.10.006 24268819 PMC3880623

[B34] Lüscher C, Slesinger PA (2010) Emerging roles for G protein-gated inwardly rectifying potassium (GIRK) channels in health and disease. Nat Rev Neurosci 11:301–315. 10.1038/nrn2834 20389305 PMC3052907

[B35] Madisen L, Zwingman TA, Sunkin SM, Oh SW, Zariwala HA, Gu H, Ng LL, Palmiter RD, Hawrylycz MJ, Jones AR, Lein ES, Zeng H (2010) A robust and high-throughput Cre reporting and characterization system for the whole mouse brain. Nat Neurosci 13:133–140. 10.1038/nn.2467 20023653 PMC2840225

[B36] Maren S (2001) Neurobiology of Pavlovian fear conditioning. Annu Rev Neurosci 24:897–931. 10.1146/annurev.neuro.24.1.897 11520922

[B37] Maren S, Phan KL, Liberzon I (2013) The contextual brain: implications for fear conditioning, extinction and psychopathology. Nat Rev Neurosci 14:417–428. 10.1038/nrn3492 23635870 PMC5072129

[B38] Marron Fernandez de Velasco E, Carlblom N, Xia Z, Wickman K (2017) Suppression of inhibitory G protein signaling in forebrain pyramidal neurons triggers plasticity of glutamatergic neurotransmission in the nucleus accumbens core. Neuropharmacology 117:33–40. 10.1016/j.neuropharm.2017.01.021 28131769 PMC5386829

[B39] McDonald AJ (1998) Cortical pathways to the mammalian amygdala. Prog Neurobiol 55:257–332. 9643556 10.1016/s0301-0082(98)00003-3

[B40] McDonald AJ, Mott DD (2017) Functional neuroanatomy of amygdalohippocampal interconnections and their role in learning and memory. J Neurosci Res 95:797–820. 10.1002/jnr.23709 26876924 PMC5094901

[B41] Nader K, Majidishad P, Amorapanth P, LeDoux JE (2001) Damage to the lateral and central, but not other, amygdaloid nuclei prevents the acquisition of auditory fear conditioning. Learn Mem 8:156–163. 10.1101/lm.38101 11390635 PMC311372

[B42] Namburi P, Beyeler A, Yorozu S, Calhoon GG, Halbert SA, Wichmann R, Holden SS, Mertens KL, Anahtar M, Felix-Ortiz AC, Wickersham IR, Gray JM, Tye KM (2015) A circuit mechanism for differentiating positive and negative associations. Nature 520:675–678. 10.1038/nature14366 25925480 PMC4418228

[B43] Nielen MM, den Boer JA, Smid HG (2009) Patients with obsessive-compulsive disorder are impaired in associative learning based on external feedback. Psychol Med 39:1519–1526. 10.1017/S0033291709005297 19243647

[B44] Nonaka A, Toyoda T, Miura Y, Hitora-Imamura N, Naka M, Eguchi M, Yamaguchi S, Ikegaya Y, Matsuki N, Nomura H (2014) Synaptic plasticity associated with a memory engram in the basolateral amygdala. J Neurosci 34:9305–9309. 10.1523/JNEUROSCI.4233-13.2014 25009263 PMC6608355

[B45] Onishi BK, Xavier GF (2010) Contextual, but not auditory, fear conditioning is disrupted by neurotoxic selective lesion of the basal nucleus of amygdala in rats. Neurobiol Learn Mem 93:165–174. 10.1016/j.nlm.2009.09.007 19766728

[B46] Pape HC, Pare D (2010) Plastic synaptic networks of the amygdala for the acquisition, expression, and extinction of conditioned fear. Physiol Rev 90:419–463. 10.1152/physrev.00037.2009 20393190 PMC2856122

[B47] Rainnie DG, Asprodini EK, Shinnick-Gallagher P (1993) Intracellular recordings from morphologically identified neurons of the basolateral amygdala. J Neurophysiol 69:1350–1362. 10.1152/jn.1993.69.4.1350 8492168

[B48] Romanski LM, Clugnet MC, Bordi F, LeDoux JE (1993) Somatosensory and auditory convergence in the lateral nucleus of the amygdala. Behav Neurosci 107:444–450. 8329134 10.1037//0735-7044.107.3.444

[B49] Roth BL (2016) DREADDs for neuroscientists. Neuron 89:683–694. 10.1016/j.neuron.2016.01.040 26889809 PMC4759656

[B50] Rozeske RR, Valerio S, Chaudun F, Herry C (2015) Prefrontal neuronal circuits of contextual fear conditioning. Genes Brain Behav 14:22–36. 10.1111/gbb.12181 25287656

[B51] Sah P, Faber ES, Lopez De Armentia M, Power J (2003) The amygdaloid complex: anatomy and physiology. Physiol Rev 83:803–834. 10.1152/physrev.00002.2003 12843409

[B52] Smith Y, Paré D (1994) Intra-amygdaloid projections of the lateral nucleus in the cat: PHA-L anterograde labeling combined with postembedding GABA and glutamate immunocytochemistry. J Comp Neur 342:232–248. 10.1002/cne.903420207 7911130

[B53] Sonner JM, Cascio M, Xing Y, Fanselow MS, Kralic JE, Morrow AL, Korpi ER, Hardy S, Sloat B, Eger EI 2nd, Homanics GE (2005) Alpha 1 subunit-containing GABA type A receptors in forebrain contribute to the effect of inhaled anesthetics on conditioned fear. Mol Pharmacol 68:61–68. 10.1124/mol.104.009936 15833735

[B54] Sosulina L, Meis S, Seifert G, Steinhäuser C, Pape HC (2006) Classification of projection neurons and interneurons in the rat lateral amygdala based upon cluster analysis. Mol Cell Neurosci 33:57–67. 10.1016/j.mcn.2006.06.005 16861000

[B55] Spampanato J, Polepalli J, Sah P (2011) Interneurons in the basolateral amygdala. Neuropharmacology 60:765–773. 10.1016/j.neuropharm.2010.11.006 21093462

[B56] Taniguchi H, He M, Wu P, Kim S, Paik R, Sugino K, Kvitsiani D, Fu Y, Lu J, Lin Y, Miyoshi G, Shima Y, Fishell G, Nelson SB, Huang ZJ (2011) A resource of Cre driver lines for genetic targeting of GABAergic neurons in cerebral cortex. Neuron 71:995–1013. 10.1016/j.neuron.2011.07.026 21943598 PMC3779648

[B57] Taylor JR, Torregrossa MM (2015) Pharmacological disruption of maladaptive memory. Handb Exp Pharmacol 228:381–415. 10.1007/978-3-319-16522-6_13 25977090

[B58] Tipps ME, Raybuck JD, Buck KJ, Lattal KM (2014) Delay and trace fear conditioning in C57BL/6 and DBA/2 mice: issues of measurement and performance. Learn Mem 21:380–393. 10.1101/lm.035261.114 25031364 PMC4105718

[B59] Tsien JZ, Chen DF, Gerber D, Tom C, Mercer EH, Anderson DJ, Mayford M, Kandel ER, Tonegawa S (1996) Subregion- and cell type-restricted gene knockout in mouse brain. Cell 87:1317–1326. 8980237 10.1016/s0092-8674(00)81826-7

[B60] Vereczki VK, Veres JM, Müller K, Nagy GA, Rácz B, Barsy B, Hájos N (2016) Synaptic organization of perisomatic GABAergic inputs onto the principal cells of the mouse basolateral amygdala. Front Neuroanat 10:20. 10.3389/fnana.2016.00020 27013983 PMC4779893

[B61] Veres JM, Nagy GA, Vereczki VK, Andrási T, Hájos N (2014) Strategically positioned inhibitory synapses of axo-axonic cells potently control principal neuron spiking in the basolateral amygdala. J Neurosci 34:16194–16206. 10.1523/JNEUROSCI.2232-14.2014 25471561 PMC6608493

[B62] Veres JM, Nagy GA, Hajos N (2017) Perisomatic GABAergic synapses of basket cells effectively control principal neuron activity in amygdala networks. eLife 6:e20721. 10.7554/eLife.20721 28060701 PMC5218536

[B63] Victoria NC, Marron Fernandez de Velasco E, Ostrovskaya O, Metzger S, Xia Z, Kotecki L, Benneyworth MA, Zink AN, Martemyanov KA, Wickman K (2016) G protein-gated K+ channel ablation in forebrain pyramidal neurons selectively impairs fear learning. Biol Psychiatry 80:796–806. 10.1016/j.biopsych.2015.10.004 26612516 PMC4862939

[B64] Wolff SB, Gründemann J, Tovote P, Krabbe S, Jacobson GA, Müller C, Herry C, Ehrlich I, Friedrich RW, Letzkus JJ, Lüthi A (2014) Amygdala interneuron subtypes control fear learning through disinhibition. Nature 509:453–458. 10.1038/nature13258 24814341

[B65] Woodruff AR, Sah P (2007) Inhibition and synchronization of basal amygdala principal neuron spiking by parvalbumin-positive interneurons. J Neurophysiol 98:2956–2961. 10.1152/jn.00739.2007 17715201

[B66] Yiu AP, Mercaldo V, Yan C, Richards B, Rashid AJ, Hsiang HL, Pressey J, Mahadevan V, Tran MM, Kushner SA, Woodin MA, Frankland PW, Josselyn SA (2014) Neurons are recruited to a memory trace based on relative neuronal excitability immediately before training. Neuron 83:722–735. 10.1016/j.neuron.2014.07.017 25102562

[B67] Zelikowsky M, Hersman S, Chawla MK, Barnes CA, Fanselow MS (2014) Neuronal ensembles in amygdala, hippocampus, and prefrontal cortex track differential components of contextual fear. J Neurosci 34:8462–8466. 10.1523/JNEUROSCI.3624-13.2014 24948801 PMC4061389

[B68] Zhou Y, Won J, Karlsson MG, Zhou M, Rogerson T, Balaji J, Neve R, Poirazi P, Silva AJ (2009) CREB regulates excitability and the allocation of memory to subsets of neurons in the amygdala. Nat Neurosci 12:1438–1443. 10.1038/nn.2405 19783993 PMC2783698

